# 5,5′-(Disulfanedi­yl)bis­(1-methyl-1*H*-tetra­zole)

**DOI:** 10.1107/S1600536811029862

**Published:** 2011-07-30

**Authors:** Ai-Yun Cui, Jing-Lin Liu

**Affiliations:** a Faculty of Chemistry, Northeast Normal University, 130024 Changchun, Jilin, People’s Republic of China; b Institute of Functional Nano and Soft Materials (FUNSOM), Soochow University, 215123 Suzhou, Jiangsu, People’s Republic of China; c College of Chemistry and Chemical Engineering, Inner Mongolia University for the Nationalities, 028042 Tongliao, Inner Mongolia, People’s Republic of China

## Abstract

In the title mol­ecule, C_4_H_6_N_8_S_2_, two tetra­zole rings linked by a disulfide bridge form a dihedral angle of 71.32 (7)° [C—S—S—C torsion angle = −80.51 (10)°]. In the crystal, strong inter­molecular π–π inter­actions between the tetra­zole rings [centroid–centroid distance = 3.285 (3) Å] link pairs of mol­ecules into centrosymmetric dimers. Weak inter­molecular C—H⋯N hydrogen bonds further link these dimers, related by translation in the [100] direction, into columns.

## Related literature

For related structures, see: Kim *et al.* (2003 ▶); Brito *et al.* (2007 ▶); Tamilselvi & Mugesh (2010 ▶). For their use as ligands in transition-metal coordination chemistry, see: She *et al.* (2006 ▶); Carballo *et al.* (2009 ▶); Wang *et al.* (2010 ▶); Aromi *et al.* (2011 ▶).
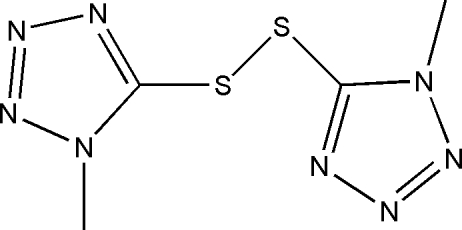


## Experimental

### 

#### Crystal data

C_4_H_6_N_8_S_2_
*M* *_r_* = 230.29
Monoclinic, 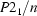

*a* = 6.3232 (3) Å
*b* = 8.1625 (3) Å
*c* = 18.3623 (7) Å
β = 98.906 (2)°
*V* = 936.31 (7) Å^3^
*Z* = 4
Mo *K*α radiation
μ = 0.54 mm^−1^
*T* = 296 K
0.40 × 0.20 × 0.20 mm

#### Data collection

Bruker APEXII CCD area-detector diffractometer
Absorption correction: multi-scan (*SADABS*; Bruker, 2005 ▶) *T*_min_ = 0.812, *T*_max_ = 0.899
8223 measured reflections
1606 independent reflections
1527 reflections with *I* > 2σ(*I*)
*R*_int_ = 0.019

#### Refinement

*R*[*F* ^2^ > 2σ(*F* ^2^)] = 0.036
*wR*(*F* ^2^) = 0.106
*S* = 1.09
1606 reflections
127 parameters
H-atom parameters constrained
Δρ_max_ = 0.38 e Å^−3^
Δρ_min_ = −0.35 e Å^−3^

### 

Data collection: *APEX2* (Bruker, 2005 ▶); cell refinement: *APEX2*; data reduction: *SAINT* (Bruker, 2005 ▶); program(s) used to solve structure: *SHELXTL* (Sheldrick, 2008 ▶); program(s) used to refine structure: *SHELXTL*; molecular graphics: *SHELXTL*; software used to prepare material for publication: *SHELXTL*.

## Supplementary Material

Crystal structure: contains datablock(s) global, I. DOI: 10.1107/S1600536811029862/cv5132sup1.cif

Structure factors: contains datablock(s) I. DOI: 10.1107/S1600536811029862/cv5132Isup2.hkl

Supplementary material file. DOI: 10.1107/S1600536811029862/cv5132Isup3.cml

Additional supplementary materials:  crystallographic information; 3D view; checkCIF report

## Figures and Tables

**Table 1:** Hydrogen-bond geometry (Å, °)

*D*—H⋯*A*	*D*—H	H⋯*A*	*D*⋯*A*	*D*—H⋯*A*
C4—H4*C*⋯N6^i^	0.96	2.61	3.518 (4)	158

Symmetry code: (i) 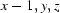.

**Fig. 1.**
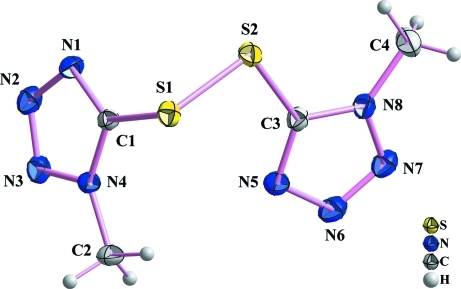
View of (I) showing the atomic labeling and 30% probability displacement ellipsoids.

## supplementary crystallographic information

### Comment

In recent years, the interesting coordination chemistry and increasingly biomedical properties of complexes derived from bis(1-methyl-1*H*-tetrazol) disulfide ligand have received much attention (Kim *et al.*, 2003; She *et al.*, 2006; Brito *et al.*, 2007; Carballo *et al.*, 2009; Tamilselvi & Mugesh, 2010; Wang *et al.*, 2010). Herein we report the synthesis and crystal structure of the title compound,  (I).

In (I) (Fig. 1), the bond lengths and angles are normal and correspond to those observed in the related compounds (Kim *et al.*, 2003; Brito *et al.*, 2007; Tamilselvi & Mugesh, 2010) The dihedral angle between the two tetrazol rings is 71.32 (7) °. The S—S bond length is 2.0474 (8) Å. The C—S—S—C torsion angle of -80.51 (10) ° compares well with that of -79.71 (10) ° reported by Tamilselvi & Mugesh (2010). The C—S—S—C torsion angle in two bis-tetrazol disulfides reported by Kim *et al.* (2003) and Brito *et al.* (2007) are 81.54 (5) ° and 80.42 (6) °, respectively.

In the crystal structure, strong intermolecular π—π interaction between the tetrazole rings [centroid-centroid distance of 3.285 (3) Å] link two molecules into centrosymmetric dimer. Weak intermolecular C—H···N hydrogen bonds (Table 1) link further these dimers related by translation in [100] into columns.

### Experimental

A water solution (5 ml) of Fe(NO_3_)_3_ (0.25 mmol) was added slowly to the water solution (15 ml) of 1-methyl-5-mercaptotetrazole (0.10 mmol). The reaction mixture was stirred at room temperature for 3 h. The solvent was removed and the solid product recrystallized from ethanol. After six days, the colourless crystals suitable for X-ray diffraction  were obtained.

### Refinement

All H atoms were placed in idealized positions and refined using a riding model (C—H = 0.96 Å) with *U*_iso_(H) = 1.5 *U*_eq_(C).

### Crystal data

**Table:**

C_4_H_6_N_8_S_2_	*F*(000) = 472
*M**_r_* = 230.29	*D*_x_ = 1.634 Mg m^−^^3^
Monoclinic, *P*2_1_/*n*	Mo *K*α radiation, λ = 0.71073 Å
Hall symbol: -P 2yn	Cell parameters from 9988 reflections
*a* = 6.3232 (3) Å	θ = 2.5–40.5°
*b* = 8.1625 (3) Å	µ = 0.54 mm^−^^1^
*c* = 18.3623 (7) Å	*T* = 296 K
β = 98.906 (2)°	Block, colourless
*V* = 936.31 (7) Å^3^	0.40 × 0.20 × 0.20 mm
*Z* = 4	

### Data collection

**Table:**

Bruker APEXII CCD area-detector diffractometer	1606 independent reflections
Radiation source: fine-focus sealed tube	1527 reflections with *I* > 2σ(*I*)
graphite	*R*_int_ = 0.019
phi and ω scans	θ_max_ = 25.0°, θ_min_ = 2.7°
Absorption correction: multi-scan (*SADABS*; Bruker, 2005)	*h* = −7→5
*T*_min_ = 0.812, *T*_max_ = 0.899	*k* = −9→9
8223 measured reflections	*l* = −21→21

### Refinement

**Table:**

Refinement on *F*^2^	Primary atom site location: structure-invariant direct methods
Least-squares matrix: full	Secondary atom site location: difference Fourier map
*R*[*F*^2^ > 2σ(*F*^2^)] = 0.036	Hydrogen site location: inferred from neighbouring sites
*wR*(*F*^2^) = 0.106	H-atom parameters constrained
*S* = 1.09	*w* = 1/[σ^2^(*F*_o_^2^) + (0.0575*P*)^2^ + 0.6856*P*] where *P* = (*F*_o_^2^ + 2*F*_c_^2^)/3
1606 reflections	(Δ/σ)_max_ < 0.001
127 parameters	Δρ_max_ = 0.38 e Å^−^^3^
0 restraints	Δρ_min_ = −0.35 e Å^−^^3^

### Special details

**Table:**

Geometry. All e.s.d.'s (except the e.s.d. in the dihedral angle between two l.s. planes)are estimated using the full covariance matrix. The cell e.s.d.'s are takeninto account individually in the estimation of e.s.d.'s in distances, anglesand torsion angles; correlations between e.s.d.'s in cell parameters are onlyused when they are defined by crystal symmetry. An approximate (isotropic)treatment of cell e.s.d.'s is used for estimating e.s.d.'s involving l.s. planes.
Refinement. Refinement of *F*^2^ against ALL reflections. The weighted *R*-factor *wR* andgoodness of fit *S* are based on *F*^2^, conventional *R*-factors *R* are basedon *F*, with *F* set to zero for negative *F*^2^. The threshold expression of*F*^2^ > σ(*F*^2^) is used only for calculating *R*-factors(gt) *etc*. and isnot relevant to the choice of reflections for refinement. *R*-factors basedon *F*^2^ are statistically about twice as large as those based on *F*, and *R*-factors based on ALL data will be even larger.

### Fractional atomic coordinates and isotropic or equivalent isotropic displacement parameters (Å^2^)

**Table:**

	*x*	*y*	*z*	*U*_iso_*/*U*_eq_	
S1	0.21952 (9)	0.05034 (7)	0.39949 (3)	0.0367 (2)	
S2	0.13427 (9)	0.20613 (8)	0.47738 (3)	0.0427 (2)	
N2	0.2328 (4)	0.3565 (3)	0.24114 (12)	0.0461 (5)	
N1	0.1217 (3)	0.2818 (2)	0.28933 (11)	0.0393 (5)	
C1	0.2630 (3)	0.1878 (2)	0.32986 (11)	0.0296 (4)	
N4	0.4531 (3)	0.2035 (2)	0.30732 (10)	0.0348 (4)	
N6	0.7056 (3)	0.3555 (3)	0.55018 (13)	0.0484 (5)	
N5	0.5698 (3)	0.2755 (3)	0.49736 (12)	0.0448 (5)	
C3	0.3824 (4)	0.2790 (3)	0.52098 (12)	0.0351 (5)	
N8	0.4035 (3)	0.3574 (2)	0.58550 (10)	0.0386 (4)	
N3	0.4312 (3)	0.3089 (3)	0.25086 (11)	0.0430 (5)	
C2	0.6534 (4)	0.1195 (4)	0.33142 (15)	0.0503 (6)	
H2B	0.6382	0.0491	0.3722	0.075*	
H2C	0.7640	0.1985	0.3465	0.075*	
H2A	0.6908	0.0551	0.2915	0.075*	
N7	0.6092 (3)	0.4054 (3)	0.60292 (12)	0.0485 (5)	
C4	0.2441 (5)	0.3978 (4)	0.63164 (16)	0.0627 (8)	
H4C	0.1080	0.3535	0.6101	0.094*	
H4A	0.2330	0.5146	0.6355	0.094*	
H4B	0.2858	0.3518	0.6799	0.094*	

### Atomic displacement parameters (Å^2^)

**Table:**

	*U*^11^	*U*^22^	*U*^33^	*U*^12^	*U*^13^	*U*^23^
S1	0.0401 (4)	0.0366 (3)	0.0343 (3)	−0.0056 (2)	0.0089 (2)	0.0033 (2)
S2	0.0329 (3)	0.0600 (4)	0.0367 (3)	−0.0085 (2)	0.0095 (2)	−0.0078 (2)
N2	0.0515 (12)	0.0450 (11)	0.0406 (11)	−0.0020 (9)	0.0028 (9)	0.0106 (9)
N1	0.0344 (10)	0.0417 (10)	0.0408 (11)	0.0010 (8)	0.0024 (8)	0.0035 (8)
C1	0.0268 (10)	0.0327 (10)	0.0293 (10)	−0.0033 (8)	0.0041 (8)	−0.0015 (8)
N4	0.0300 (9)	0.0411 (10)	0.0337 (10)	−0.0034 (7)	0.0065 (8)	0.0021 (7)
N6	0.0352 (11)	0.0539 (12)	0.0547 (13)	−0.0054 (9)	0.0024 (10)	0.0047 (10)
N5	0.0364 (11)	0.0549 (12)	0.0439 (11)	−0.0038 (9)	0.0088 (9)	0.0025 (9)
C3	0.0346 (12)	0.0382 (12)	0.0326 (11)	−0.0034 (9)	0.0051 (9)	0.0065 (9)
N8	0.0379 (10)	0.0422 (10)	0.0348 (10)	−0.0049 (8)	0.0028 (8)	0.0005 (8)
N3	0.0453 (12)	0.0498 (11)	0.0348 (10)	−0.0081 (9)	0.0090 (9)	0.0071 (8)
C2	0.0261 (11)	0.0749 (18)	0.0508 (14)	0.0049 (11)	0.0086 (10)	0.0060 (13)
N7	0.0397 (11)	0.0527 (12)	0.0503 (12)	−0.0071 (9)	−0.0023 (10)	0.0009 (10)
C4	0.0513 (16)	0.092 (2)	0.0472 (15)	−0.0110 (16)	0.0141 (13)	−0.0212 (15)

### Geometric parameters (Å, °)

**Table:**

S1—C1	1.754 (2)	N5—C3	1.324 (3)
S1—S2	2.0474 (8)	C3—N8	1.335 (3)
S2—C3	1.752 (2)	N8—N7	1.349 (3)
N2—N3	1.299 (3)	N8—C4	1.452 (3)
N2—N1	1.357 (3)	C2—H2B	0.9600
N1—C1	1.317 (3)	C2—H2C	0.9600
C1—N4	1.337 (3)	C2—H2A	0.9600
N4—N3	1.338 (3)	C4—H4C	0.9600
N4—C2	1.448 (3)	C4—H4A	0.9600
N6—N7	1.287 (3)	C4—H4B	0.9600
N6—N5	1.359 (3)		
			
C1—S1—S2	101.53 (7)	C3—N8—C4	130.1 (2)
C3—S2—S1	102.49 (8)	N7—N8—C4	121.8 (2)
N3—N2—N1	111.26 (18)	N2—N3—N4	106.28 (18)
C1—N1—N2	104.84 (19)	N4—C2—H2B	109.5
N1—C1—N4	109.43 (19)	N4—C2—H2C	109.5
N1—C1—S1	127.98 (17)	H2B—C2—H2C	109.5
N4—C1—S1	122.48 (16)	N4—C2—H2A	109.5
C1—N4—N3	108.17 (18)	H2B—C2—H2A	109.5
C1—N4—C2	130.23 (19)	H2C—C2—H2A	109.5
N3—N4—C2	121.46 (19)	N6—N7—N8	106.3 (2)
N7—N6—N5	111.64 (19)	N8—C4—H4C	109.5
C3—N5—N6	104.7 (2)	N8—C4—H4A	109.5
N5—C3—N8	109.2 (2)	H4C—C4—H4A	109.5
N5—C3—S2	128.82 (19)	N8—C4—H4B	109.5
N8—C3—S2	121.87 (17)	H4C—C4—H4B	109.5
C3—N8—N7	108.06 (19)	H4A—C4—H4B	109.5
			
C1—S1—S2—C3	−80.51 (10)	S1—S2—C3—N5	17.9 (2)
N3—N2—N1—C1	0.9 (3)	S1—S2—C3—N8	−165.44 (17)
N2—N1—C1—N4	−0.2 (2)	N5—C3—N8—N7	0.3 (3)
N2—N1—C1—S1	−176.46 (16)	S2—C3—N8—N7	−176.89 (16)
S2—S1—C1—N1	−65.6 (2)	N5—C3—N8—C4	177.5 (3)
S2—S1—C1—N4	118.62 (17)	S2—C3—N8—C4	0.3 (4)
N1—C1—N4—N3	−0.5 (2)	N1—N2—N3—N4	−1.2 (3)
S1—C1—N4—N3	175.98 (15)	C1—N4—N3—N2	1.0 (2)
N1—C1—N4—C2	−176.1 (2)	C2—N4—N3—N2	177.1 (2)
S1—C1—N4—C2	0.4 (3)	N5—N6—N7—N8	0.3 (3)
N7—N6—N5—C3	−0.1 (3)	C3—N8—N7—N6	−0.4 (3)
N6—N5—C3—N8	−0.2 (3)	C4—N8—N7—N6	−177.8 (2)
N6—N5—C3—S2	176.80 (18)		

### Hydrogen-bond geometry (Å, °)

**Table:**

*D*—H···*A*	*D*—H	H···*A*	*D*···*A*	*D*—H···*A*
C4—H4C···N6^i^	0.96	2.61	3.518 (4)	158.

Symmetry codes: (i) *x*−1, *y*, *z*.
